# The brain lipidomes of subcortical ischemic vascular dementia and mixed dementia^☆^

**DOI:** 10.1016/j.neurobiolaging.2014.02.025

**Published:** 2014-10

**Authors:** Sin Man Lam, Yuting Wang, Xinrui Duan, Markus R. Wenk, Raj N. Kalaria, Christopher P. Chen, Mitchell K.P. Lai, Guanghou Shui

**Affiliations:** a State Key Laboratory of Molecular Developmental Biology, Institute of Genetics and Developmental Biology, Chinese Academy of Sciences, Beijing, China; b Department of Biological Sciences, National University of Singapore, Singapore; c NGS Graduate School for Integrative Sciences and Engineering, National University of Singapore, Singapore; d Life Science Institute, National University of Singapore, Singapore; e Department of Biochemistry, National University of Singapore, Singapore; f Institute for Ageing and Health, Newcastle University, Campus for Ageing and Vitality, Newcastle upon Tyne, UK; g Department of Pharmacology, Yong Loo Lin School of Medicine, National University of Singapore, Singapore; h Memory, Aging and Cognition Centre, National University Health System, Singapore

**Keywords:** Lipidomics, Subcortical ischemic vascular dementia, Mixed dementia, Alzheimer's disease, Sphingolipids, Phospholipids, Mass spectrometry

## Abstract

Despite its importance as the leading cause of vascular dementia, the primary pathogenic mechanisms in subcortical ischemic vascular dementia (SIVD) have remained elusive. Because of the lack of approved therapeutic agents for SIVD, there is a pressing need to identify novel therapeutic targets. Comparative lipidomic analyses of SIVD and mixed dementia (i.e., SIVD and Alzheimer's disease, MixD) may also confer new insights pertaining to the possible interaction between neurodegenerative and vascular mechanisms in the pathogenesis of dementia. Liquid chromatography coupled to mass spectrometry was used to comprehensively analyze the lipidomes of white and gray matter from the temporal cortex of nondemented controls, SIVD, and MixD subjects. Detailed molecular profiles highlighted the pathologic relevance of gray matter sphingolipid fatty acyl chain heterogeneity in dementia. In addition, the levels of sulfatides and lysobisphosphatidic acids were progressively increased in the temporal cortex gray matter from control to SIVD to MixD. White matter phospholipid profiles indicated possible adaptive mechanisms (i.e., increased unsaturation) to chronic ischemia in SIVD and elevated membrane degradation in MixD.

## 1 Introduction

Subcortical ischemic vascular dementia (SIVD) is considered a more clinically homogenous subtype of vascular dementia (VaD) and represents a leading cause of vascular cognitive impairment and dementia, arising primarily from small-artery disease and hypoperfusion (Román et al., 2002). SIVD is predominantly the result of both complete infarcts (lacunar infarcts and microinfarcts) and rarefaction in the deep cerebral white matter, also termed white matter lesions (Román et al., 2002). Although SIVD accounts substantially for cases of cognitive decline in the elderly individuals, it often remains undiagnosed (Román et al., 2002). Given the relatively long prodromal period for the development of dementia, a therapeutic window may be available for medical intervention before the onset of frank cognitive and behavioral symptoms if accurate diagnosis could be made at the preclinical or pre-dementia stage (Exalto et al., 2012; Frisoni et al., 2010). The identification of reliable markers that could delineate the early pathogenic events in dementia could therefore alleviate the escalating public health burden associated with the disease (O'Brien et al., 2003).

In the last decade, considerable progress had been achieved in understanding the Alzheimer's disease (AD), both in terms of elucidating disease pathogenesis and developing treatment strategies, which is in stark contrast to the dearth of novel data pertaining to VaD (O'Brien et al., 2003). Currently, there are no approved medications available for treating VaD, and treatment is usually confined to controlling associated vascular risk factors such as hypertension and dyslipidemia (Moretti et al., 2011). Furthermore, cholinesterase inhibitors and memantine designated for the treatment of AD displayed limited, if any, benefit to VaD patients (Simonsen et al., 2012). Thus, there is a pressing need to identify novel therapeutic agents effective for VaD treatment. Studies that address the role of brain lipid aberrations in VaD pathogenesis have been particularly scarce, especially in the recent decade. Perturbation of brain lipid homeostasis in VaD had been previously investigated by Wallin et al. (1989) chiefly using thin-layer chromatography. The group reported substantial reductions in cerebrosides and sulfatides, and to a smaller extent, cholesterols and phospholipids in the white matter of VaD and AD patients, respectively (Wallin et al., 1989). Nonetheless, systematic profiling of the comprehensive brain lipidome of VaD patients is still lacking and therefore requires comprehensive instrumental analyses. Indeed, molecular species within the same lipid class could be altered to varying extent or even in a reciprocal manner depending on the level of unsaturation and/or fatty acyl chain lengths, as had been previously demonstrated by Chan et al. (2012) in postmortem AD brain tissues and AD mouse models. Thus, the elucidation of a precise molecular lipid signature that defines SIVD is prerequisite for deciphering the mechanistic roles of lipids in dementia stemming from vascular-related pathologies. The clinically homogenous nature of SIVD, as well as its status as the leading cause of vascular-related dementia in the elderly individuals (Román et al., 2002), renders it a suitable candidate for investigating the role of lipids in VaD pathogenesis.

Mounting evidence indicate the frequent coexistence of brain vascular lesions and neurodegenerative changes in cognitively impaired individuals (Petrovitch et al., 2005), and it was found that these disorders were often juxtaposed in most population samples (Snowdon et al., 1997). For instance, in the Honolulu-Asia Aging Study, neuropathological examination of demented individuals revealed an appreciable overlap, as well as autonomous contributions of AD pathology and vascular infarcts to the development of dementia (Petrovitch et al., 2005). Indeed, these distinct categories of brain pathologies could possibly produce synergistic effects culminating in the clinical expression of cognitive impairment and dementia, known as mixed dementia (MixD) (O'Brien et al., 2003), defined originally by Hachinski, (1990) as “Alzheimer's disease and cerebral infarcts contributing to the dementia”. Possibly, vascular damages could reduce the threshold for the clinical manifestation of AD (Esiri et al., 1999; Exalto et al., 2012). Accumulating data have shown that the occurrence of pure VaD is rare compared with the mixed phenotype (Enciu et al., 2011). Moreover, vascular risk factors including hypertension, hyperlipidemia, diabetes mellitus, and the metabolic syndrome predispose individuals to both AD and SIVD (de la Torre, 2010). Indeed, vasculopathy (and the resultant ischemia) had long been proposed as an alternative etiology of AD apart from the broadly accepted amyloid hypothesis (de la Torre, 2004; Pluta and Amek, 2008; Scheibel et al., 1989). In fact, in his seminal work in 1907, Alzheimer also noted cerebrovascular aberrations of the endothelial walls, particularly for small vessels in the brains of AD patients, which received considerably less attention compared with amyloid-beta aggregates and neurofibrillary tangles that now constitute the hallmark features of AD (Pluta and Amek, 2008). The co-occurrence of disease phenotypes implies that vascular defects and neurodegenerative changes may interact on several levels. Elucidating the molecular details of such interactions, as well as the respective contributions of vascular and Alzheimer's pathology to cognitive deficits in MixD might therefore help identify the primary pathogenic mechanism in AD per se.

In this study, we report the comprehensive lipidomic profiling of the white matter and gray matter from the temporal cortex of patients with SIVD and MixD, respectively, and in comparison to age-matched, nondemented controls using high-performance liquid-chromatography coupled to mass spectrometry (HPLC/MS) as the principal analytical platform. To our knowledge, this is the first lipidomic study to systematically characterize the distinct brain lipid molecular signatures of SIVD and MixD patients. Association between brain lipid profiles and neuropathological parameters for dementia (i.e., neuritic plaques and neurofibrillary tangles) were also investigated by comparing variations in the lipidomic patterns as a function of increasing senile plaque densities and tau pathology. This study (1) identifies novel molecular therapeutic targets for SIVD; and (2) confers new insights pertaining to the interactions between vascular and neurodegenerative lipid pathology in contributing to dementia.

## 2 Methods

### 2.1 Subjects and tissue processing

Postmortem frozen brain tissues from SIVD, MixD, and age-matched normal control subjects (Supplementary Table S1) were obtained from the Newcastle Brain Tissue Resource, Institute for Ageing and Health, Newcastle University. Informed consent was obtained from the guardians of the patients before donation of brain tissues and approval was granted by local research ethics committees (National University Health System, Singapore and Newcastle upon Tyne Hospitals Trust, UK). For this study, we assessed samples of both gray and white matter from the temporal lobe (Brodmann area 21). We focused on the temporal lobe because medial temporal lobe atrophy is a common finding in dementia and our recent study suggested a vascular basis for neurodegeneration (Gemmell et al., 2012). The temporal lobe is also relatively free of large infarcts (Kalaria et al., 2004).

Final classification of subjects was assigned based on established neuropathological diagnostic criteria. Briefly, hematoxylin–eosin staining was used for assessment of structural integrity and infarcts, Nissl and Luxol fast blue staining for cellular pattern and myelin loss, Bielschowsky silver impregnation for “CERAD” rating of neuritic plaques (Mirra et al., 1991) and tau immunohistochemistry for “Braak” staging of neurofibrillary tangles (Braak and Braak, 1991). A diagnosis of SIVD was made when there were multiple or cystic infarcts, lacunae, microinfarcts, and small vessel disease, and Braak stage < III (Kalaria et al., 2004). The diagnosis of MixD was assigned when there was evidence of significant AD pathology, namely Braak stage V-VI, and moderate-severe vascular pathology. Vascular pathology was graded according to the scoring system described previously (Deramecourt et al., 2012). Control subjects had no clinical evidence of dementia, neurologic, or psychiatric disease. The age-matched controls had no history of cognitive or psychiatric symptoms and died from nonneurologic causes such as bronchopneumonia, pulmonary embolism, and cardiac failure. At postmortem, control brains were Braak stage III or below and did not meet diagnostic criteria for AD (Mirra et al., 1991) or SIVD (Kalaria et al., 2004). Furthermore, tissue samples from controls were determined not to have sufficient pathology to reach the threshold to ascertain a diagnosis for dementia.

### 2.2 Lipid extraction

Frozen tissues were inactivated with 900 μL of chloroform:methanol (1:2) containing 10% deionized H_2_O. Tissue samples were cut into fine pieces using micro-scissors on dry ice, and methanol was used to thoroughly wash the micro-scissors between samples. Samples were homogenized and incubated at 1200 rpm, 4 °C, for 1 hour in a thermomixer. At the end of the incubation, 400 μL of deionized H_2_O and 300 μL of chloroform were added and vortexed. Samples were then centrifuged at 12000 rpm, 4 °C, for 5 minutes. Lower organic phase was extracted. Second extraction was carried out by adding 50 μL of 1 M hydrochloric acid and 500 μL of chloroform. Extracted organic fractions were pooled and dried speed-valco (Thermo Savant, Milford, USA). Samples were stored at −80 °C until further analysis.

### 2.3 Mass spectrometric analysis

Polar lipids were analyzed on an Agilent 1200 HPLC system coupled with an Applied Biosystem Triple Quadrupole/Ion Trap mass spectrometers 3200 Qtrap as described previously (Shui et al., 2011a,b). PC-14:0/14:0, PE-14:0/14:0, PS-14:0/14:0, PA-17:0/17:0, PG-14:0/14:0, PI-34:1/d31, LPC-C20:0, LPE-C17:0, C8-GluCer, C17-Cer, C14-LBPA, C12-SM, C12-SL were obtained from Avanti Polar Lipids (Alabaster, AL, USA). Dioctanoyl phosphatidylinositol (PI, 16:0-PI) was used for lysophosphatidylinositol quantitation and obtained from Echelon Biosciences, Inc (Salt Lake City, UT, USA). Glycerol lipids triacylglycerides (TAG) and diacyglycerides (DAG) were analyzed using a modified protocol of reverse-phase HPLC/ESI/MS described previously (Shui et al., 2010). Separation of lipids aforementioned was carried out on a Phenomenex Kinetex 2.6μ-C18 column (i.d. 4.6 × 100 mm) using an isocratic mobile phase chloroform:methanol:0.1 M ammonium acetate (100:100:4) at a flow rate of 150 μL/min for 17 minutes. Levels of TAG were calculated relative to the spiked d5-TAG 48:0 internal standard (CDN isotopes), while DAG species were quantified using 4 ME 16:0 Diether DG as an internal standard (Avanti Polar Lipids, Alabaster, AL, USA). Free cholesterol and cholesterol esters were analyzed as described previously with corresponding d6-cholesterol and d6-C18 cholesterol ester (CDN isotopes) as internal standards (Shui et al., 2011a,b).

## 3 Statistical analysis

Molar fractions of individual lipid classes and species were used for statistical analyses. One-way analysis of variance with post hoc Tukey test was performed to compare the changes in lipid profiles between control, SIVD, and MixD. To evaluate the associations between neuropathological parameters (i.e., CERAD plaque score, Braak staging) and lipid changes, subjects were classified into separate groups based on CERAD plaque score (Group 1: none/sparse; Group 2: moderate/frequent) and Braak staging (Group 1: 0–IV; Group 2: V–VI), respectively. Student *t* test was used to compare the changes in lipid profiles between the 2 groups within each clinical classification. For all bar graphs, error bars represent standard errors of the mean. For all cases, #*p* < 0.10; *p < 0.05; **p < 0.01; ***p < 0.001.

## 4 Results

A total of 334 distinct lipid species from 17 lipid subclasses including neutral lipids such as cholesteryl esters, free cholesterols, DAG, TAG; sphingolipids including ceramides (Cer), galactosylceramides (GalCer), glucosylceramides (GluCer), sphingomyelins (SM), ganglioside mannoside 3 (GM3), and sulfatides (SL); as well as phospholipid subclasses of phosphatidylcholines (PC), phosphatidylethanolamines (PE), phosphatidylglycerols (PG), phosphatidylinositols (PI), phosphatidylserines (PS), phosphatidic acids (PA), and lysobisphosphatidic acids (LBPA) were analyzed. The purity of the white and gray matter obtained in this study was verified based on their respective molecular compositions of plasmalogen phosphatidylethanolamine species (pPE) (Han et al., 2001) (Supplementary Fig. S1). Interestingly, global alterations in lipid profiles for SIVD patients were concentrated in the white matter (Supplementary Fig. S2), whereas that for MixD (i.e., AD + VaD) were mainly localized to the gray matter (Supplementary Fig. S3). These observations were aligned with the neuropathophysiology of the respective diseases, because SIVD is largely characterized by vascular lesions of the cerebral white matter (Román et al., 2002; Tomimoto, 2011), while AD essentially entails a dysfunction of chemical synapses coupled with degeneration of the hippocampal neurons (Dubois et al., 2010; Selkoe, 2002), both of which are mainly found in the gray matter of the brain tissues.

### 4.1 Overall reductions in sphingolipids in SIVD white matter

**Fig. 1:**
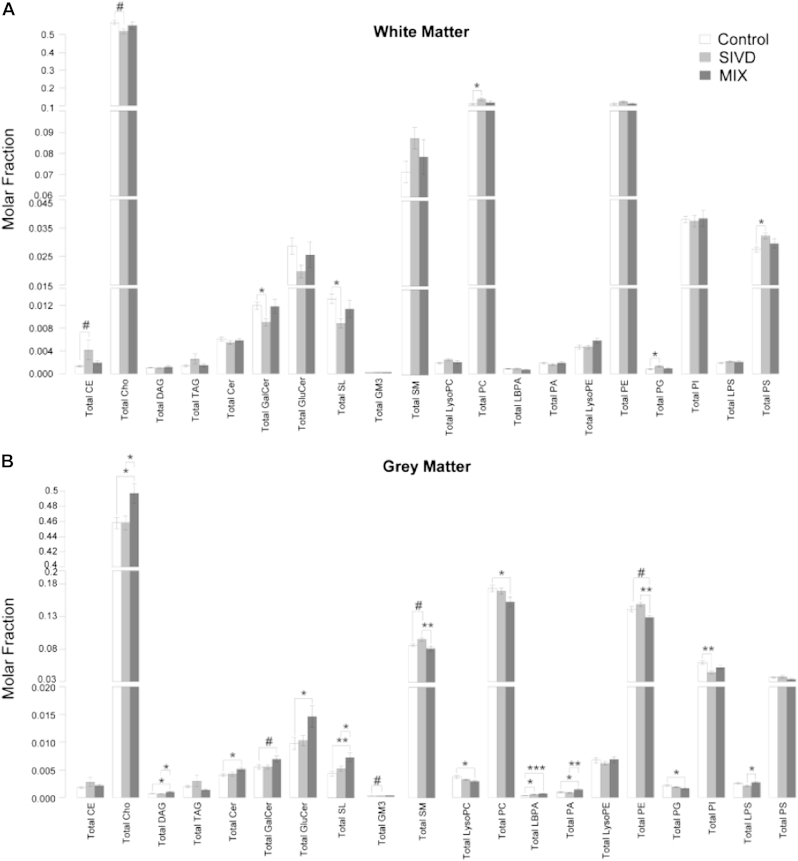
Changes in the levels of individual lipid subclasses amongst control (n = 14), SIVD (n = 11), and MixD (n = 10) for (A) white matter and (B) gray matter. Lipid classes were expressed as mean molar fractions with error bars indicating mean ± standard errors. Abbreviations: MixD, mixed dementia; SIVD, subcortical ischemic vascular dementia.

**Fig. 2:**
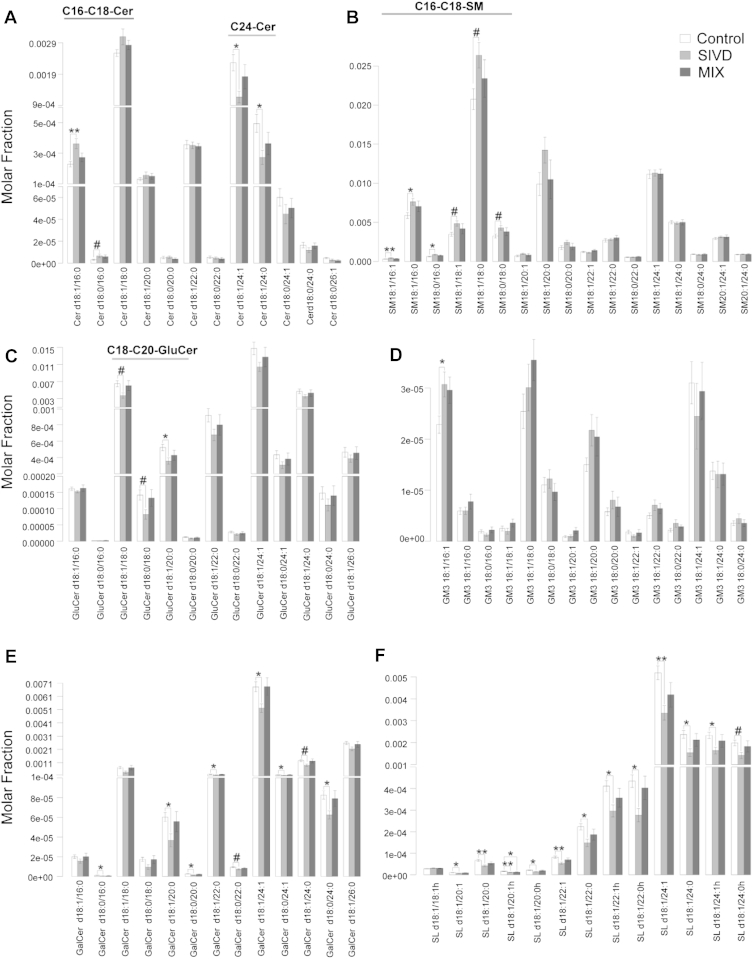
Comparative lipid profiles of sphingolipids including (A) Cer, (B) SM, (C) GluCer, (D) GM3, (E) GalCer, and (F) SL in the white matter of control (n = 14), SIVD (n = 11), and MixD (n = 10). Lipid species were expressed as mean molar fractions with error bars indicating mean ± standard errors. Abbreviations: Cer, ceramides; GalCer, galactosylceramides; GM3, ganglioside mannoside 3; GluCer, glucosylceramides; MixD, mixed dementia; SIVD, subcortical ischemic vascular; SL, sulfatides; SM, sphingomyelins.

Significant reductions in the classes of GalCer and SL (*p* < 0.05) were observed in SIVD white matter, in corroboration with Wallin et al. (1989), who observed decreases in cerebrosides and SL in VaD. In addition, Cer and GluCer were also decreased albeit not reaching statistical significance, while total SM was increased. Also in agreement with previous study based on thin-layer chromatography (Wallin et al., 1989), total GM3 levels were not significantly altered (Fig. 1A). Molecular profiles of the sphingolipidome showed that Cer species comprising short-chain fatty acyls (16–18C) were elevated, while the levels of very long chain (VLC)-Cer (24C) were specifically reduced (*p* < 0.05) (Fig. 2A). Parallel increases in short-chain SM with the corresponding fatty acyl chain lengths (*p* < 0.05) (Fig. 2B) were observed, accompanied by diminished levels of C18-GluCer (Fig. 2C). Notably, GM3 18:1/16:1 exhibited ∼25% increase in SIVD compared with control (*p* < 0.05) (Fig. 2D). On the other hand, individual species of GalCer and SL were all consistently reduced in SIVD white matter (Fig. 2E and F). The specific increases in short-chain Cer and SM in SIVD white matter suggested that excess short-chain Cer were probably channeled into SM, resulting in diminished levels of other downstream glycosphingolipids including GluCer, GalCer, and SL with the corresponding fatty acyl moieties. On the other hand, the overall reductions in glycosphingolipids containing VLC-fatty acyls might have resulted from the attenuated biosynthesis of VLC-Cer upstream.

### 4.2 Changes in phospholipids in SIVD white matter

**Fig. 3:**
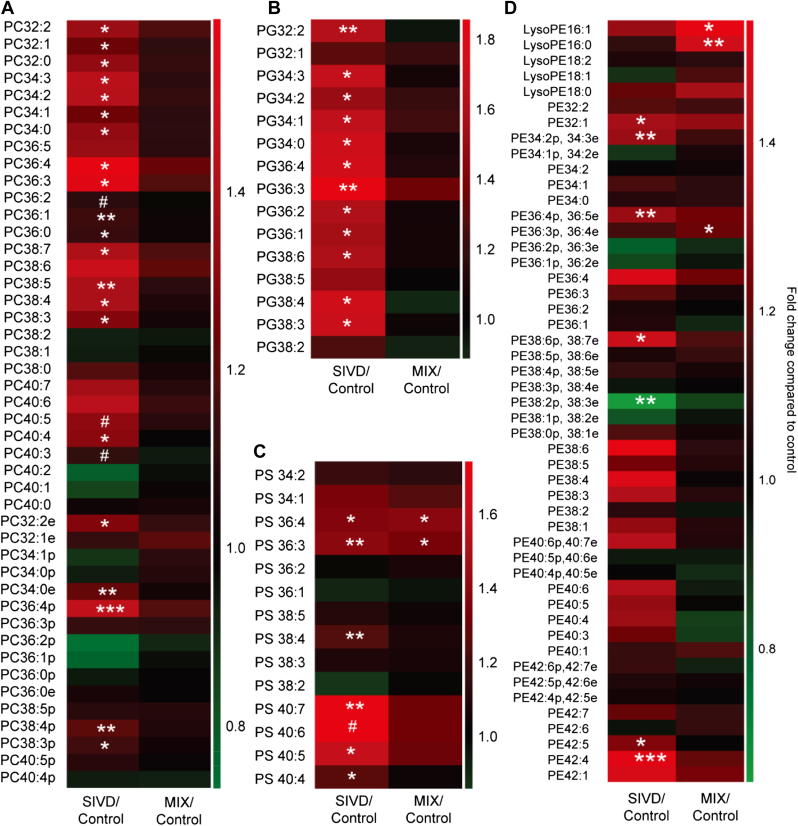
Heatmaps illustrate the fold change in individual phospholipid species in the white matter of SIVD and MixD compared against control from the subclasses of (A) PC, (B) PG, (C) PS, and (D) PE. Abbreviations: PC, phosphatidylcholines; PE, phosphatidylethanolamines; PG, phosphatidylglycerols; PS, phosphatidylserines.

Contrary to the overall reductions in the white matter sphingolipidome, appreciable increases were observed in numerous phospholipid classes including PC, PS, and PG (*p* < 0.05) (Fig. 1B). Notably, individual species of PC, PS, and PG were also predominantly elevated in SIVD with statistical significance (*p* < 0.05) (Fig. 3A–C). The trend was remarkably striking for PG with ∼1.5-fold increase, for which all individual species were also consistently increased (Fig. 3B). Also notable is that the levels of several polyunsaturated PS, such as PS 36:3, PS 36:4, PS 38:4, PS 40:7, PS 40:5, and PS 40:4, were specifically augmented in SIVD (*p* < 0.05) (Fig. 3C). While total PE did not change appreciably (Fig. 1A), molecular compositions of pPE were altered according to their levels of unsaturation. Notably, PE 36:2p, 36:3e, the most abundant species in nondemented controls, were reduced substantially in SIVD while PE 40:6p, 40:7e was increased with marginal significance (*p* < 0.10) (Fig. 3D). On another note, most diacyl-PE species were increased in SIVD (Fig. 3D).

### 4.3 Enhanced sphingolipids levels in MixD gray matter

**Fig. 4:**
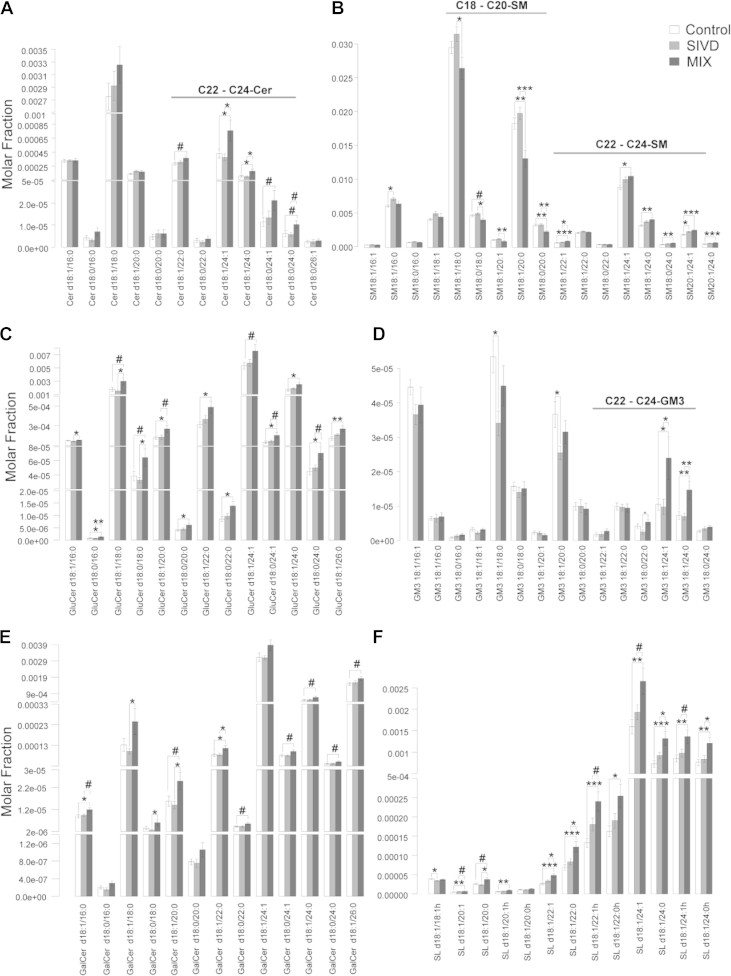
Comparative lipid profiles of sphingolipids including (A) Cer, (B) SM, (C) GluCer, (D) GM3, (E) GalCer, and (F) SL in the gray matter of control (n = 14), SIVD (n = 11), and MixD (n = 10). Lipid species were expressed as mean molar fractions with error bars indicating mean ± standard errors. Abbreviations: Cer, ceramides; GalCer, galactosylceramides; GM3, ganglioside mannoside 3; GluCer, glucosylceramides; MixD, mixed dementia; SIVD, subcortical ischemic vascular; SL, sulfatides; SM, sphingomyelins.

The levels of various sphingolipid classes were predominantly increased in MixD gray matter (Fig. 4). With the exception of SM and GM3, which exhibited changes dependent on fatty acyl heterogeneity (see the following), total Cer (*p* < 0.05), GluCer (*p* < 0.05), GalCer (*p* < 0.10), and SL (*p* < 0.05) were all appreciably increased in MixD gray matter (Fig. 1B). Detailed molecular profiles indicated that VLC-Cer (22–24C) were significantly enriched (Fig. 4A), with parallel increases in VLC-SM containing the corresponding fatty acyls (Fig. 4B). In contrast, SM species comprising shorter fatty acid residues (18–20C) were significantly reduced in MixD gray matter (Fig. 4B). The reciprocal changes in SM based on fatty acyl chain lengths were in agreement with previous observations in postmortem prefrontal cortex but surprisingly opposite to that in the entorhinal cortex of AD human brains (Chan et al., 2012). While individual species of GluCer were all consistently increased (Fig. 4C), short-chain GM3 (16–20C) were reduced compared with control, albeit not reaching statistical significance (Fig. 4D). On the other hand, VLC-GM3 displayed remarkable increases in MixD, with ∼2-fold increase observed for GM3 18:1/24:1 and GM3 18:1/24:0 (*p* < 0.05) (Fig. 4D), which coincides with the reported selective enrichment of VLC-GM3 in human AD entorhinal cortex (Chan et al., 2012). In addition, all individual species of GalCer and SL were consistently increased in MixD gray matter compared with controls (Fig. 4E and F). In accordance with our observed increase in SL with neuropathology, Lim et al. (2013) had previously reported enhanced SL levels in aged apolipoprotein E (APOE)-ε4 knock-in mice compared with their ε3 counterparts under high-fat-high-cholesterol diet, and that individual SL species were predominantly increased with aging regardless of both diet and APOE genotype (Lim et al., 2013).

Therefore, a discernible metabolic shift toward elevated levels of glycosphingolipids, instead of SM, was noted in MixD. In addition, as the levels of relatively short-chain Cer (16–20C) were only modestly increased in MixD, degradation of SM with the corresponding fatty acyls might have occurred to generate additional Cer that would serve as precursors for the biosynthesis of short-chain glycosphingolipids further downstream. Notably, long-chain SL (22–24C) exhibited progressive increases from control to SIVD to MixD (Fig. 4F), suggesting that interaction between vascular and neurodegenerative pathology in MixD might have additive effects on this specific class of lipids in the gray matter.

### 4.4 Alterations in phospholipids in MixD gray matter

**Fig. 5:**
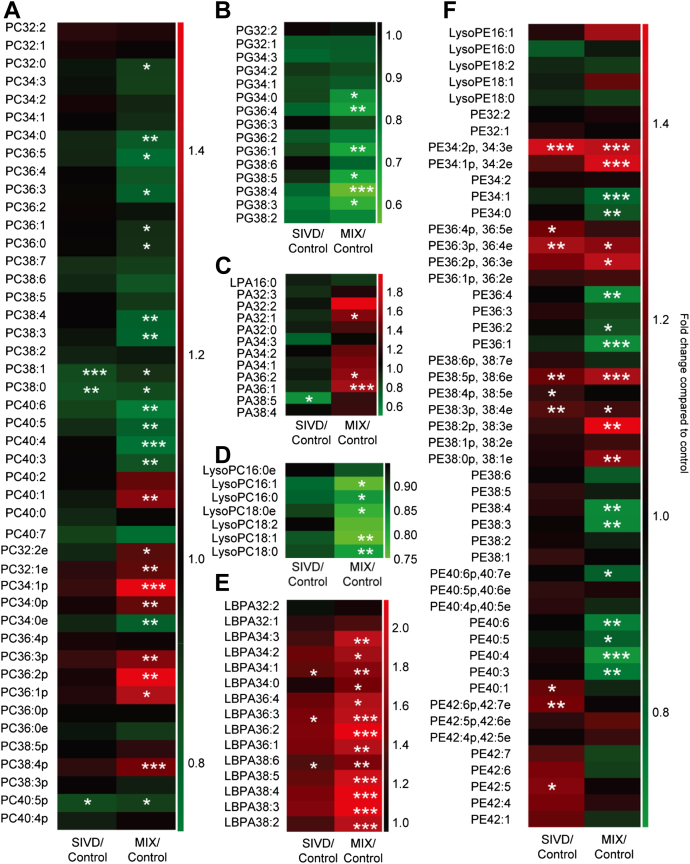
Heatmaps illustrate the fold change in individual phospholipid species in the gray matter of SIVD and MixD compared against control from the subclasses of (A) PC, (B) PG, (C) Lyso-PC, (D) PA, (E) LBPA, and (F) PE. Abbreviations: LBPA, lysobisphosphatidic acids; MixD, mixed dementia; PA, phosphatidic acids; PC, phosphatidylcholines; PE, phosphatidylethanolamines; PG, phosphatidylglycerols; PS, phosphatidylserines.

Major phospholipid classes of PC (*p* < 0.05) and PE (*p* < 0.10), as well as total PG (*p* < 0.05) were significantly reduced in MixD compared with controls (Fig. 1B). In addition, total lyso-PC (including lyso-ether PC) was decreased (*p* < 0.05) (Fig. 1B). Molecular profiling indicated that while diacyl-PC were predominantly reduced, several plasmalogen-PC (pPC) species including PC34:1p, PC34:0p, PC36:3p, PC 36:2p, PC 36:1p, and PC 38:4p were significantly enriched (*p* < 0.05) in MixD (Fig. 5A). In addition, in agreement with Chan et al. (2012), all individual PG (Fig. 5B) and lyso-PC (including lyso-ether PC) species (Fig. 5C) were consistently decreased. On another note, numerous PA species such as PA 32:1, PA 36:2, and PA 36:1 were significantly elevated (*p* < 0.05) (Fig. 5D). Notably, total LBPA were enriched by ∼1.8-fold in MixD compared with control (Fig. 1B), which is in close agreement with the reported ∼1.8-fold increase in LBPA in the entorhinal cortex of AD human brains (Chan et al., 2012). Moreover, total LBPA (Fig. 1B) and individual LBPA species (Fig. 5E) exhibited progressive increases from control to SIVD to MixD (Fig. 1B). Contrary to a previous report (Han et al., 2001) but in accordance with Chan et al. (2012), no significant changes in total pPE were observed in MixD (Supplementary Fig. S4A and B). Nonetheless, polyunsaturated pPE such as PE 40:6p, 40:7e was significantly reduced (*p* < 0.05), while less unsaturated pPE including PE 34:1p, 34:2e and PE 36:2p, 36:3e were increased (*p* < 0.05) (Fig. 5F). Indeed, an overall reduction in polyunsaturated pPE (n ≥ 4) was observed in MixD gray matter, accompanied by concomitant increases in pPE containing only 1 (*p* < 0.05), 2 (*p* < 0.01), or 3 (*p* < 0.05) double bonds in their structures (Supplementary Fig. S4D). On the other hand, total diacyl-PE (*p* < 0.01) (Supplementary Fig. S4B), as well as several individual diacyl-PE species, were significantly reduced in MixD (Fig. 5F).

### 4.5 Linking lipidomic changes to AD neuropathological parameters

**Fig. 6:**
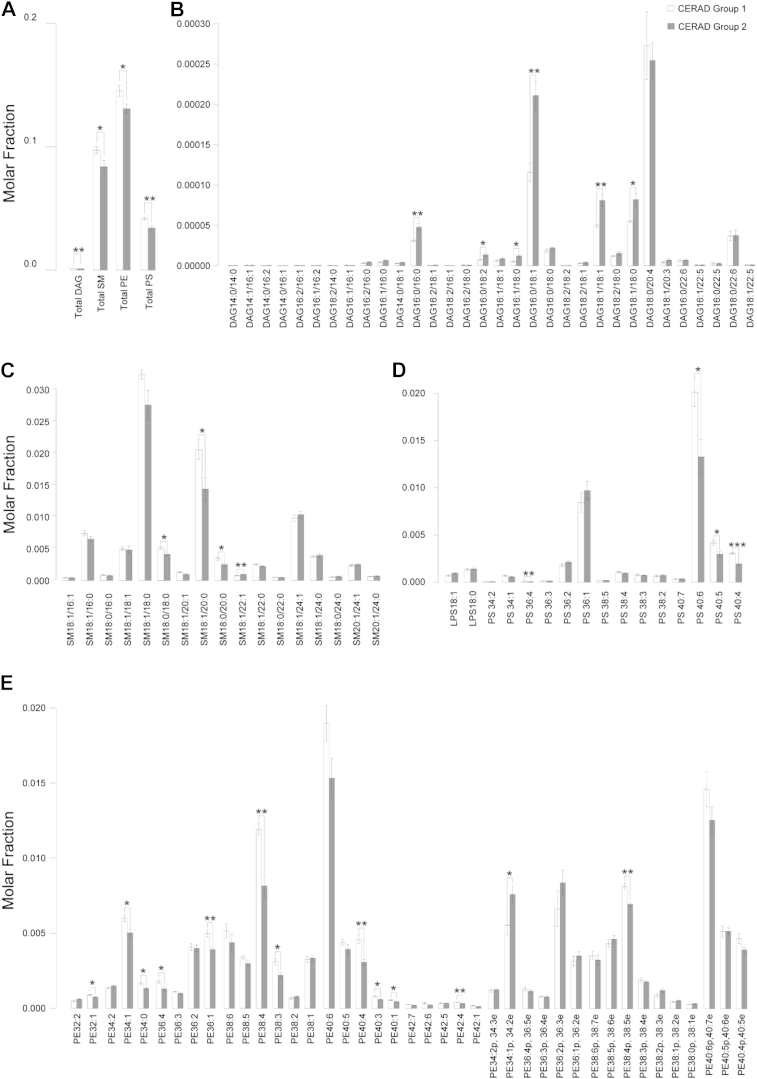
Changes in the selected classes of lipids in the gray matter as a function of increasing CERAD plaque score. The levels of (A) specific lipid subclasses and individual species from subclasses of (B) DAG, (C) PS, (D) SM, and (E) PE were significantly perturbed with increasing CERAD. Lipid species were expressed as mean molar fractions with error bars indicating mean ± standard errors. Abbreviations: DAG, diacyglycerides; PE, phosphatidylethanolamines; PS, phosphatidylserines; SM, sphingomyelins.

Significant increases in total DAG (*p* < 0.01), as well as decreases in total SM (*p* < 0.05), PE (*p* < 0.05), and PS (*p* < 0.01) were observed in the gray matter of subjects with higher CERAD plaque score (i.e., moderate and/or frequent) (Section 2.4) (Fig. 6A). Interestingly, numerous DAG species comprising palmitic (C16:0), palmitoleic (C16:1), stearic (C18:0), and oleic acids (C18:1) in their structures were markedly enriched in subjects with higher CERAD (Fig. 6B). Accordingly, total DAG (*p* < 0.05) (Fig. 1B) and similar DAG species containing saturated and/or monounsaturated fatty acids were also significantly elevated (*p* < 0.05) in MixD gray matter (Supplementary Fig. S3), which is in corroboration with previous observation in the prefrontal cortex of AD human brains (Chan et al., 2012). On the other hand, consistent with changes in SM in MixD gray matter, the reductions in relatively short-chain SM (18–20C) were also noted in subjects with higher CERAD (Fig. 6C). Remarkably, numerous polyunsaturated PS such as PS 36:4, PS 40:6, PS 40:5, and PS 40:4 were considerably reduced (*p* < 0.05) with higher CERAD (Fig. 6D). Furthermore, PS 40:6 (*p* < 0.05), PS 40:5 (*p* < 0.01), and PS 40:4 (*p* < 0.001) were also significantly decreased in MixD gray matter (Supplementary Fig. S3). In addition, changes in pPE according to the levels of unsaturation, similar to that observed in MixD gray matter, were also noted in subjects with higher CERAD. Diacyl-PE, on the other hand, was consistently reduced with higher CERAD (Fig. 6E). No associations between lipid changes in the temporal cortex and the Braak staging, however, were found in both the white and gray matter.

## 5 Discussion

While SIVD mainly affects the subcortical structures, the choice of the cortex for lipidomic analysis could potentially yield lipid changes that are etiologically linked to the subcortex on a circuit-wide basis, such as those affecting neurotransmission. Therefore, the observed lipid derangements in the cortex, instead of the damaged tissues in the subcortical regions, possibly reflect primary pathogenic events associated with the early stage of the disease. In addition, cortical regions appear less susceptible to postmortem damage and could therefore better indicate changes of pathophysiological relevance (Bowen et al., 1977).

### 5.1 Sphingolipid aberrations in SIVD indicated disruptions to myelin structural integrity

Because cerebrosides are largely confined to myelin, the considerable reductions in GluCer, GalCer, and SL in SIVD white matter suggested that the myelin sheath represents a primary site of these changes, consistent with previous observations of extensive myelin destruction with relatively minor neuronal damages in VaD (Wallin et al., 1989). It had been shown that oligodendrocytes swelling and myelin sheath aberrations occurred before (and independently of) neuronal damage in the event of ischemia (Pantoni et al., 1996). It is thus probable that under hypoperfusion in SIVD, available oxygen is preferentially channeled to preserve the functionally important neurons, leading to selective death of oligodendrocytes that also have considerably high metabolic demands (Wallin et al., 1989). Indeed, GalCer and SL are almost exclusively synthesized by oligodendrocytes (Bosio et al., 1996; Coetzee et al., 1998). This also explains the predominant localization of vascular lesions to the white matter, rather than the gray matter, of the temporal cortex observed in the present study.

Surprisingly, the sphingolipid and neutral lipid derangements observed in SIVD white matter closely resembled the brain lipid profiles of ceramides synthase 2 (CerS2)-null mice previously reported in an independent study (Ben-David et al., 2011). CerS2 preferentially uses VLC acyl-CoAs (22–24C) for biosynthesis of VLC-Cer (Laviad et al., 2008). In CerS2-null mice, reductions in C22-C24-Cer and C22-C24-SM were observed, accompanied by concomitant increases in C18-Cer and C18-SM and an overall reduction in GalCer. In addition, a marginal increase in cholesteryl esters was reported (Laviad et al., 2008). Notably, CerS2-null mice exhibited myelin degeneration and detachment from axons without appreciable neuronal loss, coupled with motor dysfunction characterized by fast myoclonic jerks and loss of posture, which were indicated to be of a subcortical origin from electroencephalogram recordings (Laviad et al., 2008). Indeed, white matter lesions and psychomotor disturbances also represent the major neuropathological and clinical manifestations of SIVD (Tomimoto, 2011).

### 5.2 Enhanced phospholipid unsaturation in SIVD as an adaptive response to chronic ischemia

The increase in phospholipid content in SIVD white matter contrasted with earlier studies reporting phospholipid catabolism in transient cerebral ischemia, which could be attributed to (1) lipid peroxidation; and/or (2) activation of hydrolytic enzymes (Yoshida et al., 1980). Nonetheless, a separate study argued against phospholipid membrane disintegration but pointed instead to an augmented level of polyunsaturated fatty acids, during ischemia (Rehncrona et al., 1982). The paradoxical increase in polyunsaturated pPE and PS under chronic hypoperfusion in SIVD might be associated with the location of white matter tissues chosen for analysis. Previous analysis had reported the predominant localization of parenchymal lesions in the frontal and parietal lobes, instead of the temporal lobe, in SIVD (Akiguchi et al., 2004). Thus, the small vessels in the temporal lobe might be less severely affected in SIVD, or only afflicted at advanced stages. Therefore, the observed enhancement in phospholipid unsaturation in the temporal cortex might be associated with “remote ischemic preconditioning” (Steiger and Hänggi, 2007), in which prolonged ischemia experienced in the frontal and parietal lobes might possibly trigger adaptive mechanisms (e.g., increase unsaturation) in the temporal cortex for protection against impending oxidative insults. Notably, ischemic preconditioning had been reported to enhance antioxidants in the brain and peripheral organs following cerebral ischemia (Glantz et al., 2005).

### 5.3 Polar lipid changes in MixD consistent with endolysosomal dysfunction and increased oxidative stress characteristic of AD molecular pathology

The global increase of sphingolipids (and LBPA) is in accord with the endolysosomal dysfunction theory for AD pathogenesis (Small and Gandy, 2006). A notable finding in our study is the reciprocal changes in SM and GM3 depending on fatty acyl heterogeneity in the MixD gray matter, which might be attributed to the critical roles of fatty acid chain lengths in mediating endocytic trafficking and lipid raft functions. Specifically, cholesterol loading was shown to elevate intracellular accumulation of long-chain SM via enhanced targeting of these species for recycling back to the plasma membrane, instead of degradation in lysosomes (Koivusalo et al., 2007), consistent with the observed increases in free cholesterols and VLC-SM in this study (Supplementary Fig. S4B). SM and gangliosides constitute important components of lipid rafts in neuronal membranes (Haughey et al., 2010). The acyl chain components of raft lipids could exert varying yet determining effects on the stability of individual membrane rafts, which may have far-reaching effects on cellular signaling and biochemistry. Indeed, similar opposing trends in GM3 based on fatty acid chain lengths had been observed in the plasma of type 2 diabetes patients and GM3 fatty acyl heterogeneity was proposed to influence lipid raft formation critical for mediating insulin receptor signaling (Shui et al., 2013). In addition, compared with short-chain GM3, the augmented levels of VLC-GM3 in gray matter might be even more detrimental to AD pathogenesis, as the longer fatty acyl chains are expected to confer stronger hydrophobic forces in overcoming the electrostatic repulsion between sialic acid residues to form ganglioside-enriched membrane clusters, previously shown to be involved in seeding amyloid-beta polymerization (Matsuzaki et al., 2010).

The overall reduction in major membrane phospholipids including PC and PE in MixD coincided with previous observations in AD, which attributed the decrease to membrane defects associated with neurodegenerative events (Nitsch et al., 1992; Pettegrew et al., 2001). In addition, the overall reduction in diacyl-PC, as well as increases in corresponding diacyl-PA and DAG species largely comprising saturated and/or monounsaturated fatty acids indicated enhanced phospholipase D activity in MixD. Indeed, phospholipase D-2 ablation had been previously shown to rescue AD-linked synaptic dysfunction and cognitive deficits in AD mouse model (Oliveira et al., 2010).

### 5.4 Contribution of vasculopathology and neuropathology to mixed dementia

While lipid changes in MixD gray matter were predominantly in accordance with AD pathology, the progressive increase in SL from control to SIVD to MixD contrasted with the previously reported SL reduction in early AD (Han et al., 2002), and in the prefrontal and entorhinal cortices of postmortem AD human brains (Chan et al., 2012). The increase in gray matter SL in MixD might thus be attributed to the additive effects of concurrent vascular lesions in addition to neurodegenerative changes. Under normal physiological condition, SL-containing APOE-associated lipoproteins are either taken up by neurons and metabolized via the endolysosomal pathway, or transported to the peripheral circulation and removed from the brain tissues via the cerebrospinal fluid (Han, 2007). A general state of hypoperfusion in brain parenchyma in SIVD and MixD could possibly impede the clearance of SL-containing lipoproteins via the cerebrospinal fluid, which might be further exacerbated in MixD because of the compromised endolysosomal pathway in neurons, leading to SL accumulation within gray matter. Indeed, endolysosomal dysfunction might have commenced in early SIVD, as evident in the progressive accumulation of LBPA in gray matter from SIVD to MixD but the effect might have been masked in SIVD because of attenuated glycosphingolipid synthesis as discussed previously.

While current lipidomic data seemed to suggest that MixD phenotype could be, to a greater extent, attributed to neuropathological aberrations instead of vasculopathology, it is nonetheless crucial to reckon that SIVD and AD might alter the brain parenchyma in a region-specific manner. As parenchymal lesions in SIVD appear predominantly in the frontal and parietal lobes (Akiguchi et al., 2004), the choice of the temporal cortex for analysis could possibly have biased the MixD lipidome toward a greater manifestation of neurodegenerative phenotype resembling AD.

### 5.5 Conclusions and future work

Lipidomics represents a useful tool that can be harnessed to determine lipid pathways that are specifically perturbed in distinct biological systems (Chan et al., 2012). Our study has therefore established a reference frame of lipids that could be targeted for advancing current diagnostic and therapeutic endeavors in the context of SIVD and MixD. In particular, based on the close resemblance in brain sphingolipid profiles of CerS2-null mice (Ben-David et al., 2011) with the anomalies in SIVD sphingolipidome reported herein, we surmise that CerS2 could be a candidate enzyme implicated in SIVD pathogenesis that warrants further investigation. Indeed, the mechanisms that connect small vessel disease with brain parenchymal lesions have remained discordant and elusive, primarily because of a lack of appropriate animal models that can convincingly reflect the pathologic aberrations observed in humans (Pantoni, 2010). Our lipidomic results suggest that CerS2-null mice could be a potentially relevant model for human SIVD that warrants future investigation.

**Fig. 7:**
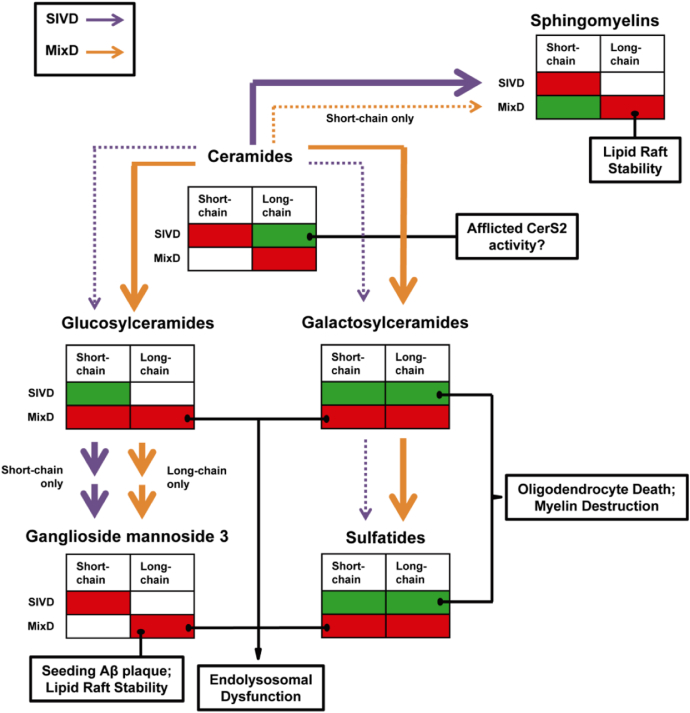
Summary of the differences in perturbed sphingolipid metabolisms between SIVD white matter and MixD gray matter. While an enhanced shunt of Cer precursors to SM synthesis was observed in SIVD white matter, an increased channeling of Cer for biosynthesis of complex glycosphingolipids downstream was noted in MixD gray matter. Bold arrows indicate increased channeling while dotted arrows indicate reduced channeling. The alterations in sphingolipid levels were subdivided based on fatty acyl chain lengths, with red indicating increase and green indicating reduction. Abbreviations: Cer, ceramides; MixD, mixed dementia; SIVD, subcortical ischemic vascular; SM, sphingomyelins.

In addition, this study underscored the critical importance of sphingolipid fatty acyl chain heterogeneity in neurodegeneration, and demonstrated the essential differences in the perturbed sphingolipid metabolisms between SIVD and MixD compared with controls. In summary, enhanced channeling of Cer precursors to SM biosynthesis was observed in SIVD white matter while biosynthesis of complex glycosphingolipids downstream of Cer was curtailed. The opposite was noted for MixD gray matter, in which a global accumulation of glycosphingolipids was observed (Fig. 7).

Our study also identified SL accumulation in the temporal cortex gray matter, which could likely be attributed to the synergistic interactions between vascular and neurodegenerative lesions co-occurring specifically in MixD, as a possible marker that could distinguish pure AD from MixD. The feasibility of sulfatides as a lipid marker to differentiate between SIVD and MixD from controls, however, needs to be further evaluated in the cerebrospinal fluid of a human cohort, although region-specific aberrations in brain lipid levels might be masked as a consequence. Furthermore, the pathogenic significance of the lipid alterations reported herein also requires verification using appropriate animal models for SIVD and MixD. Nevertheless, the present study has contributed substantially to our existing knowledge of the possible associations between brain lipid changes and the pathogenesis of dementia in a human cohort, thus providing a framework upon which future mechanistic validation can be conducted.

## Disclosure statement

The authors declare that there are no actual or potential conflicts of interest.
