# Supplementary material for: The brain lipidomes of subcortical ischemic vascular dementia and mixed dementia
Source: Neurobiol Aging. 2014 Oct;35(10):2369–81. doi: 10.1016/j.neurobiolaging.2014.02.025 (PMC4099521; doi:10.1016/j.neurobiolaging.2014.02.025)
Supplement: Supplementary Figures S1 to S5 and Supplementary Tables S1 and S2 [file mmc1.pdf]

Supplementary Fig. S1.

Comparison on the degree of unsaturation of plasmalogen PE in the white matter and grey matter. Han and colleagues had previously reported the predominant presence of pPE containing polyunsaturated fatty acids (PUFA) in the grey matter, with the unsaturated fatty acyls probably constituting essential components of signal transduction pathways. On the other hand, pPE in the white matter were found to be comparatively less unsaturated in nature (Han et al, 2001). Accordingly, the grey matter was observed to contain a significantly higher level ($p<0.001$) of polyunsaturated pPE ($n \geq 4$) than the white matter in the current study. ***$p<0.001$

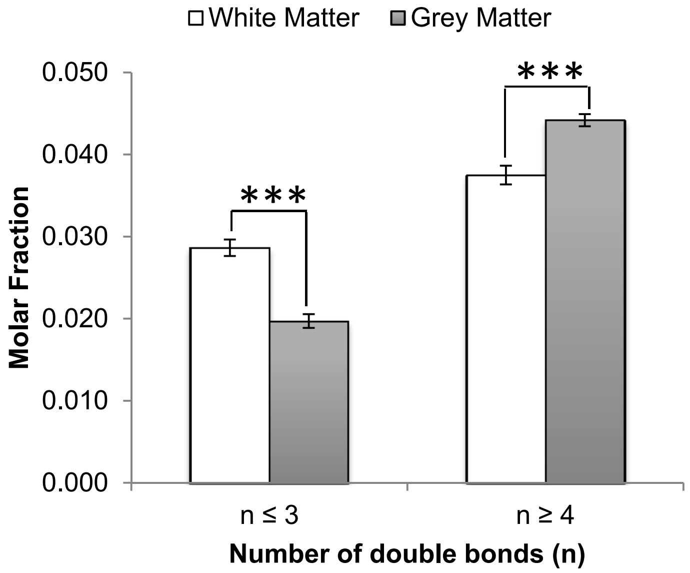

Supplementary Fig. S2.

Heatmaps illustrating the alterations in global lipid profiles of white matter in MIX and SIVD compared to controls. Z-scores were plotted and statistically significant results from One-way ANOVA with post-hoc Dunnett's test were marked. An appreciable accumulation of total CE, accompanied by a concomitant decrease in free Cho, was noted in SIVD white matter ($p<0.10$) (Fig. 1A). Molecular profiles revealed that CE 16:0, CE 18:2 and CE 18:1 were appreciably increased ($p<0.05$) in SIVD.

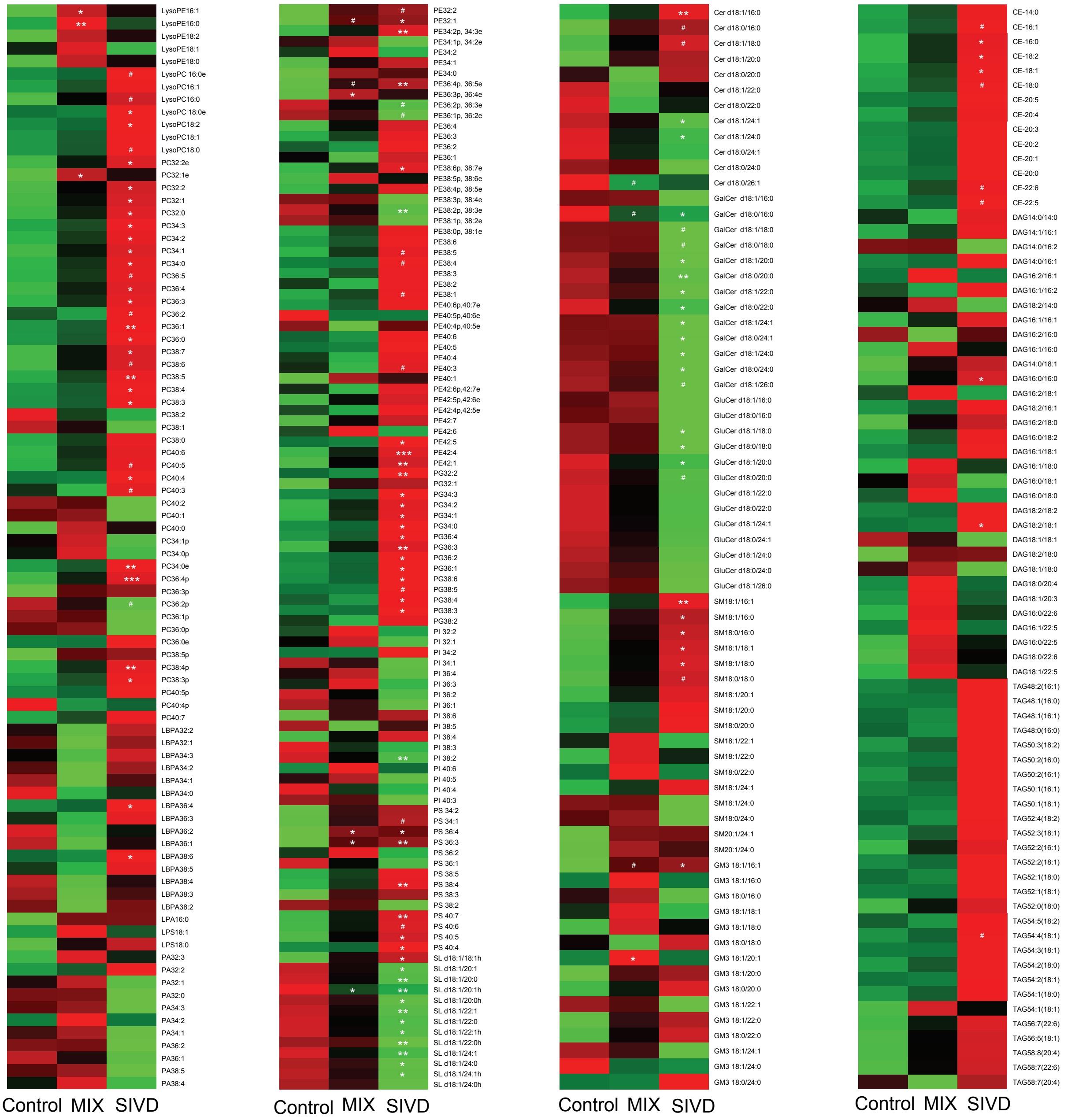

Supplementary Fig. S3.

Heatmaps illustrating the alterations in global lipid profiles of grey matter in MIX and SIVD compared to controls. Z-scores were plotted and statistically significant results from One-way ANOVA with post-hoc Dunnett's test were marked. Notably, total PI in SIVD grey matter were significantly reduced ($p<0.01$) (Fig. 1B). In addition, significant decreases in individual PI species containing three or more double bonds in their structures were also consistently observed in the grey matter.

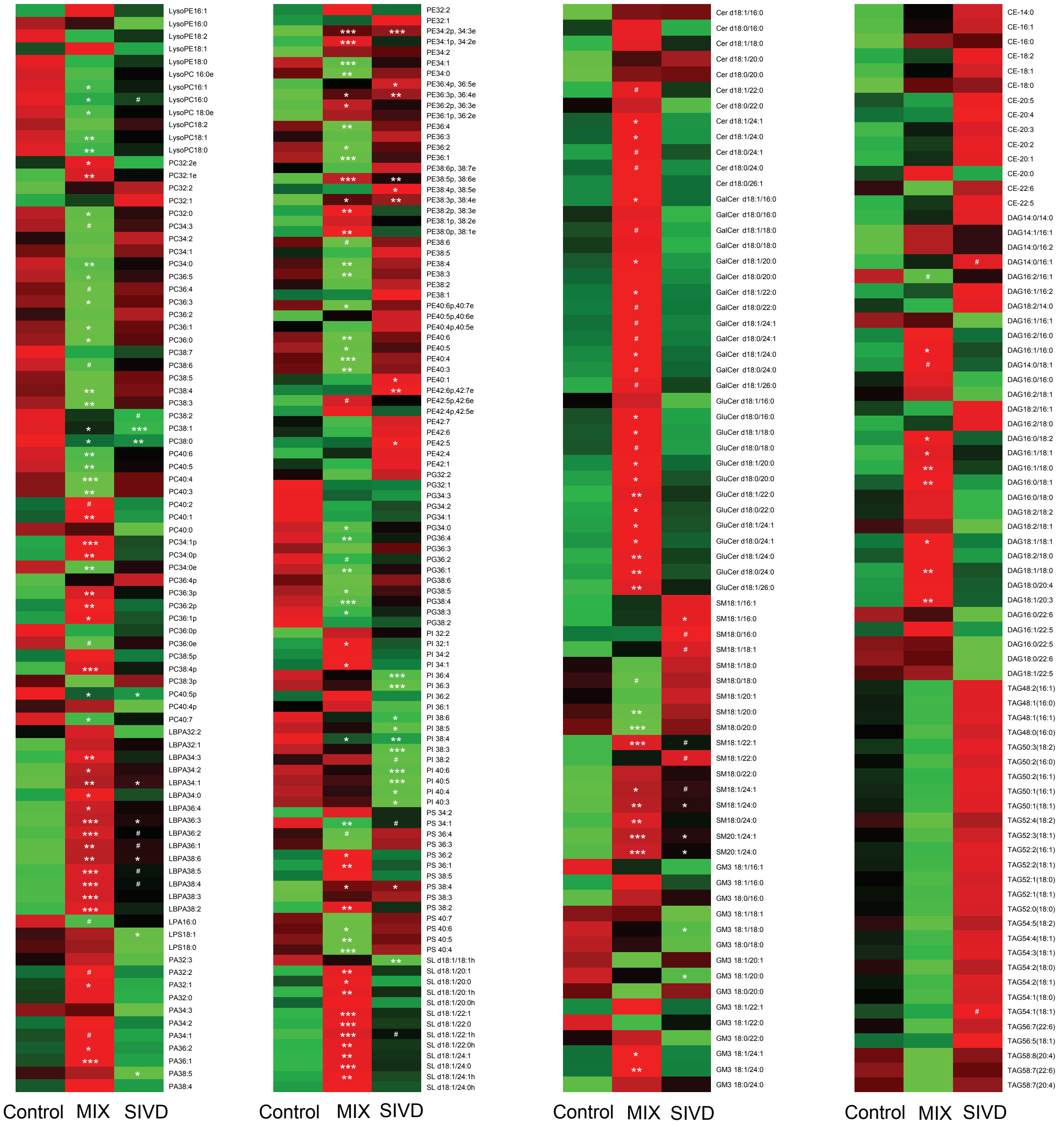

Supplementary Fig. S4.

Comparison of the levels of total plasmalogen PE and diacyl-PE in the **(A)** white matter and **(B)** grey matter of control, SIVD and MIX. Comparison of the changes in the degree of unsaturation of plasmalogen PE in the **(C)** white matter and **(D)** grey matter of control, SIVD and MIX.

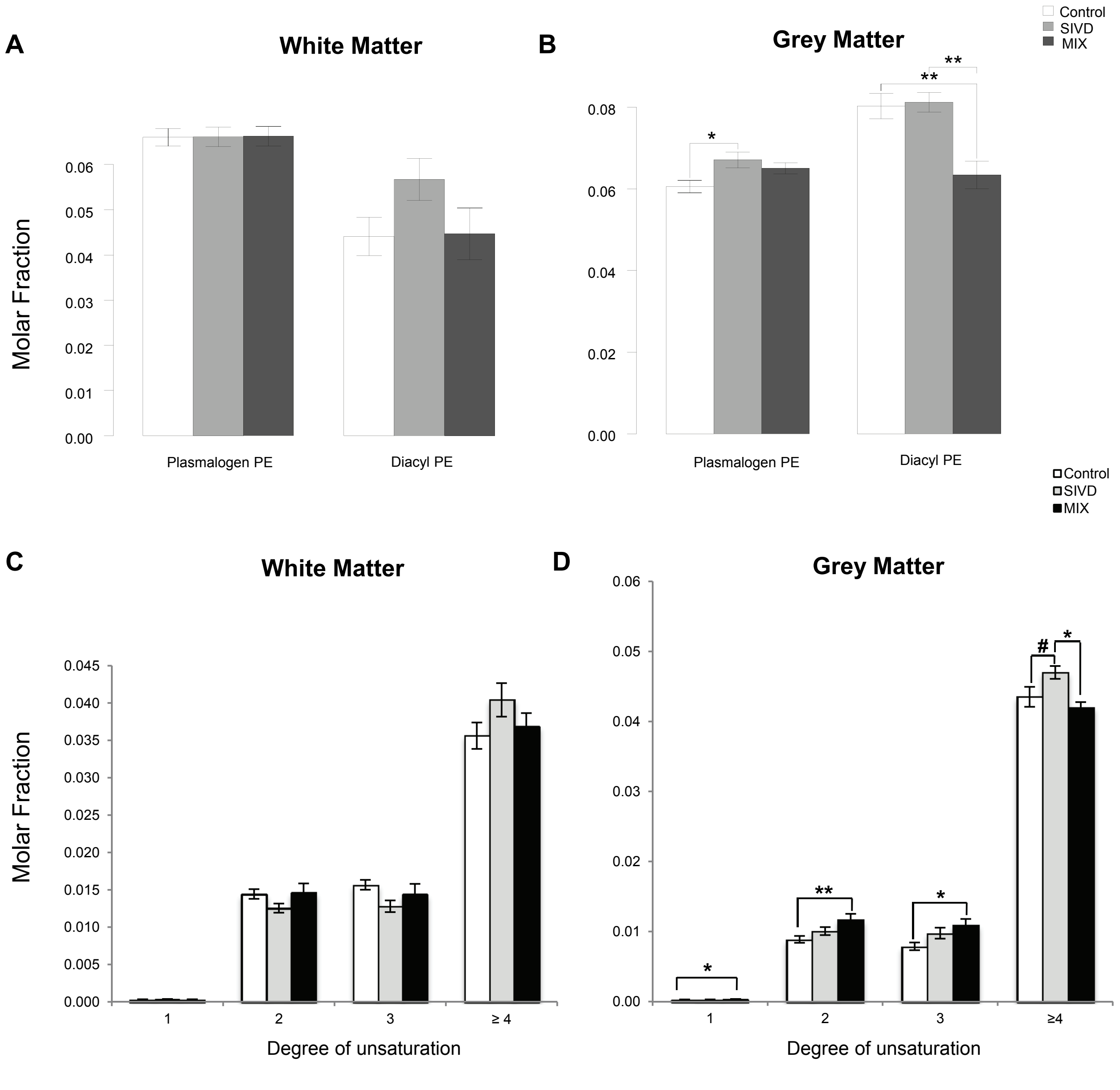

Supplementary Fig. S5.

Table displays the p-values and correlation coefficients of Spearman's correlation analyses between molar fractions of polar lipid classes and post-mortem delays **(A).** Of all lipid classes analysed, only PE levels in grey matter were significantly correlated with post-mortem delays. Scatter plot of grey matter PE illustrated that MixD individuals possessed consistently lower levels of PE than SIVD (and control) individuals with comparable post-mortem delays **(B).** Thus, the higher levels of PE observed in grey matter of SIVD (and control) compared to MixD were due to disease pathology and not post-mortem delay *per se*. The correlation analyses therefore shown that the changes in lipid classes observed between control, SIVD and MixD were not associated with post-mortem changes.

A

	White Matter		Grey Matter	
	p-value	r	p-value	r
Total Cer	0.11	0.28	0.56	0.11
Total GalactoCer	0.92	-0.02	0.51	0.12
Total GluCer	0.45	0.14	0.40	0.15
Total LysoPC	0.60	0.10	0.96	0.01
Total PC	0.71	-0.07	0.38	-0.16
Total SM	0.83	-0.04	0.64	-0.08
Total GM3	0.83	-0.04	0.16	-0.25
Total LBPA	0.92	-0.02	0.78	0.05
Total PA	0.47	-0.13	0.60	-0.10
Total LysoPE	0.23	0.21	0.22	0.22
Total PE	0.71	-0.07	0.03	-0.38
Total PG	0.66	-0.08	0.63	-0.09
Total PI	0.67	-0.08	0.07	-0.32
Total LPS	0.16	0.25	0.70	-0.07
Total PS	0.66	-0.08	0.16	-0.25
Total SL	0.90	-0.02	0.73	0.06

B

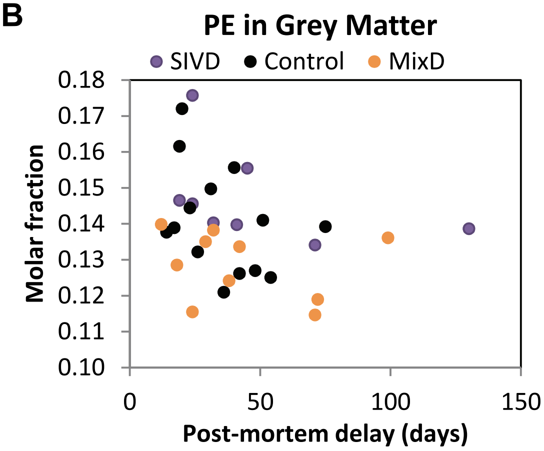

Supplementary Table S1. Demographic and disease variables in a cohort of control and dementia patients

Data are mean ± S.E.M. MixD, mixed dementia (SIVD / Alzheimer's disease); *N*, number of cases; SIVD, subcortical ischemic vascular dementia.

	Control	SIVD	MixD
N	14	11	10
Age at Death (years)	79.9 ± 2	85.3 ± 3	83.4 ± 3
Sex (Male/Female)	6M / 8F	7M / 4F	5M / 5F
Postmortem Interval (hours)	35.4 ± 5	44.8 ± 12	43.7 ± 9

Supplementary Table S2. Table displays the p-values from ANCOVA analyses controlling post-mortem delay with Tukey HSD test.

The analyses indicated that the observed changes between control, SIVD and MixD in the white and grey matter were still statistically significant after controlling post-mortem delay as a co-variable. Thus, the reported lipid changes between controls, SIVD and MixD could not be accounted for by post-mortem delays, but were reflective of differences due to disease pathology.

	White				Grey			
	**ANOVA**	**MIX - Control**	**SIVD - Control**	**SIVD - MIX**	**ANOVA**	**MIX - Control**	**SIVD - Control**	**SIVD - MIX**
**Total CE**	0.008	0.249	0.006	0.231	0.408	0.408	0.639	0.943
**Total Cho**	0.063	0.720	0.052	0.260	0.009	0.026	0.853	0.014
**Total DAG**	0.537	0.626	0.979	0.565	0.017	0.026	0.994	0.037
**Total TAG**	0.204	0.981	0.209	0.322	0.175	0.737	0.405	0.156
**Total Cer**	0.521	0.798	0.498	0.878	0.063	0.059	0.897	0.202
**Total GalactoCer**	0.056	0.998	0.068	0.101	0.052	0.091	0.950	0.075
**Total GluCer**	0.165	0.777	0.142	0.451	0.038	0.050	1.000	0.080
**Total LysoPC**	0.077	0.817	0.067	0.244	0.018	0.014	0.319	0.366
**Total PC**	0.024	0.647	0.019	0.156	0.043	0.049	0.985	0.109
**Total SM**	0.199	0.738	0.172	0.546	0.010	0.415	0.082	0.007
**Total GM3**	0.100	0.083	0.556	0.554	0.129	0.900	0.228	0.135
**Total LBPA**	0.295	0.286	0.960	0.493	0.000	0.000	0.025	0.184
**Total PA**	0.422	0.945	0.556	0.421	0.007	0.020	0.845	0.011
**Total LysoPE**	0.111	0.165	0.968	0.149	0.685	0.991	0.746	0.701
**Total PE**	0.270	0.994	0.285	0.375	0.008	0.097	0.304	0.006
**Total PG**	0.013	0.857	0.012	0.058	0.069	0.060	0.819	0.261
**Total PI**	0.880	0.972	0.868	0.960	0.021	0.254	0.017	0.414
**Total LPS**	0.452	0.753	0.429	0.859	0.132	0.813	0.279	0.124
**Total PS**	0.004	0.346	0.003	0.100	0.030	0.203	0.420	0.024
**Total SL**	0.030	0.496	0.022	0.255	0.006	0.005	0.712	0.058
